# Apoptotic Donor Cells in Transplantation

**DOI:** 10.3389/fimmu.2021.626840

**Published:** 2021-02-25

**Authors:** Irma Husain, Xunrong Luo

**Affiliations:** Department of Medicine, Duke University, Durham, NC, United States

**Keywords:** apoptosis, tolerance, transplantation, EDCI-SP, cell-based therapies

## Abstract

Despite significant advances in prevention and treatment of transplant rejection with immunosuppressive medications, we continue to face challenges of long-term graft survival, detrimental medication side effects to both the recipient and transplanted organ together with risks for opportunistic infections. Transplantation tolerance has so far only been achieved through hematopoietic chimerism, which carries with it a serious and life-threatening risk of graft versus host disease, along with variability in persistence of chimerism and uncertainty of sustained tolerance. More recently, numerous *in vitro* and *in vivo* studies have explored the therapeutic potential of silent clearance of apoptotic cells which have been well known to aid in maintaining peripheral tolerance to self. Apoptotic cells from a donor not only have the ability of down regulating the immune response, but also are a way of providing donor antigens to recipient antigen-presenting-cells that can then promote donor-specific peripheral tolerance. Herein, we review both laboratory and clinical evidence that support the utility of apoptotic cell-based therapies in prevention and treatment of graft *versus* host disease and transplant rejection along with induction of donor-specific tolerance in solid organ transplantation. We have highlighted the potential limitations and challenges of this apoptotic donor cell-based therapy together with ongoing advancements and attempts made to overcome them.

## Introduction

The use of immunosuppressive medications for transplantation has significantly decreased the incidence of acute allograft rejection, however they have had limited to no impact on chronic rejection and overall long-term graft survival (1). On the contrary, this pharmacological immunosuppression has side effects that include infections, malignancies, metabolic disease together with drug toxicities to the allograft itself. These detrimental side effects and non-specific immunosuppression can be potentially eliminated through donor-specific tolerance induction. Thus far in humans, one strategy that has been employed with encouraging results in solid organ transplantation is the use of combined kidney and hematopoietic stem cell transfers (CKHCT). This results in a state known as mixed chimerism, wherein both donor and recipient hematopoietic stem cells coexist and tolerance is achieved primarily through the central tolerance mechanism of intra-thymic deletion of donor-reactive T cells. This strategy has been successful in Human Leukocyte Antigen (2) identical transplants with the use of total lymphoid irradiation and T cell depletion for conditioning. However, in HLA-mismatched donor-recipient pairs, more aggressive conditioning was required together with administration of a higher number of donor T cells that significantly increased the risk of the life-threatening complication of graft versus host disease (3–5). In the realm of non-chimeric approaches, immunoregulatory cell-based therapies have recently come into clinical trial space as well, with the most frequently used cells being regulatory T cells (T_regs_), tolerogenic antigen-presenting-cells (APC) such as dendritic cells (DC) and regulatory macrophages, and lastly, myeloid-derived suppressor cells (MDSCs) (6–8). These cells have been used in treatment of graft-versus-host-disease (GVHD), rejection in hematopoietic stem cell transplant (HSCT) as well as tolerance induction in solid organ transplantation. However, the major challenges and hurdles of this approach include cumbersome manufacturing processes of these cells, selection of optimal timing and dose, conferring antigen specificity, and lastly, their *in vivo* instability.

Many of the aforementioned challenges encountered with the mixed chimerism approach and immunoregulatory cell therapy can be overcome with the use of apoptotic cells which can effectively deliver donor antigen while also creating an immunosuppressive milieu that promotes donor specific tolerance. Not only has this potential been utilized for tolerance induction and treatment of rejection in solid organ transplant, in HSCT it has also shown efficacy in reverting GVHD (9).

## Mechanisms

Apoptosis is essential to the maintenance of self-tolerance, thus mutations in apoptosis regulating genes such as Fas and Fas ligand (FasL) in humans as well as in mouse models have been implicated in autoimmune diseases (10, 11). Specifically, inability to effective clear dying cells can result in persistence of cellular debris which may lead to systemic autoimmunity such as systemic lupus erythematosus (12–14). Apoptotic cells attract and recruit macrophages to dying cells through “find-me” signals and facilitate engulfment through “eat-me” signals in a process known as efferocytosis (15). Efferocytosis involves four steps: recruitment, recognition, tethering and signaling and engulfment. At the onset of apoptosis, recruitment is carried out through “find‐me” signals produced by apoptotic cells. These are sensed by phagocytes which are then recruited to the site of apoptosis. The second step, involves the interaction of binding ligands (“eat-me” signals) on the surface of apoptotic cells and their receptors on the surface of macrophages. As a consequence, the cytoskeletal rearrangement within the phagocyte occurs by a Rac1‐mediated signaling pathway (16). The final step of engulfment follows this and internalization of apoptotic particles and their decomposition takes place within phagocytes.

One such “find me” signal is lysophosphatidylcholine, a lipid mediator that is produced and released from apoptotic cells and by interacting with the G2 accumulation receptor, it recruits macrophages (17). This is a G‐protein‐coupled receptor expressed in macrophages, dendritic cells, neutrophils, mast cells, T lymphocytes and B lymphocytes that is involved in regulating cell cycle, proliferation, and immunity. Its further functions are not known well, however it’s interaction with lysophosphatidylcholine possibly results in the production of chemoattractants such as monocyte chemotactic protein‐1 (MCP-1), IL‐8 and chemokine ligand 5 (CCL5) for the recruitment of monocytes, neutrophils and lymphocytes. Another “find me” signal is sphingosine‐1‐phosphate that acts on macrophages to increase erythropoietin (EPO) expression, subsequently activating the peroxisome proliferator‐activated receptor‐γ (18). This enhances the expression of numerous phagocyte receptors like MerTK, MFGE8, Gas6, and CD36, all of which play a role in promoting phagocytosis.

**Figure 1:**
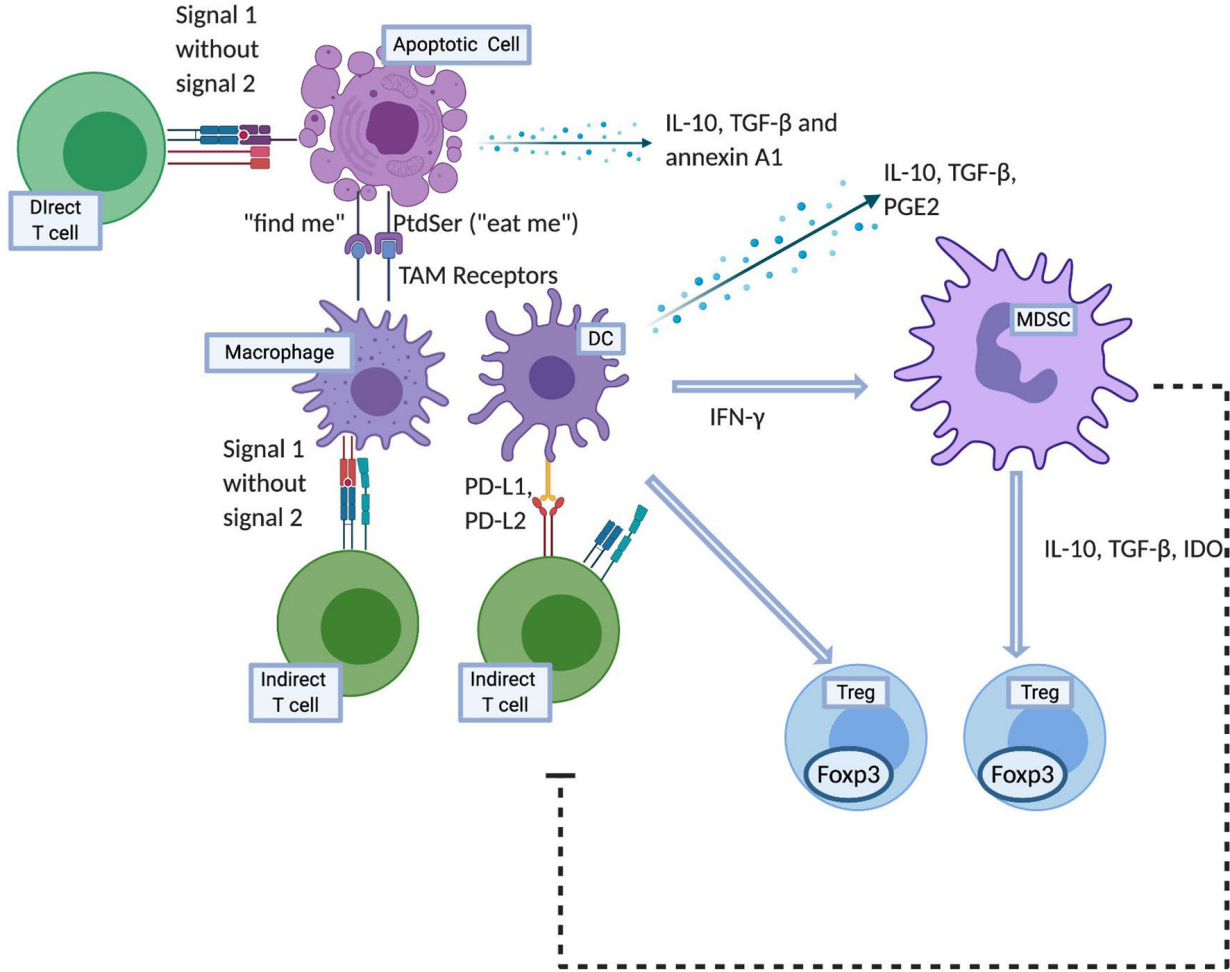
Mechanisms of apoptotic cell induced tolerance. Created with BioRender.com.

Cells express phosphatidylserine (PtdSer) on their surface when undergoing apoptosis, which then acts as an “eat‐me” signal (19, 20). Using Annexin I as a bridging molecule, PtdSer interacts with the TAM family (21) of receptors to promote phagocytosis. This TAM family are tyrosine kinases receptors for Gas6 and protein S which bind PtdSer and antagonize inflammatory cytokine production by STAT-1-dependent induction of suppressor of cytokine signaling (SOCS) proteins 1 and 3 (22, 23). Furthermore, apoptotic cell-mediated activation of Mer inhibits lipopolysaccharide (LPS) driven PI3K/AKT-dependent NF-κB activation (24). As NF-κB signaling results in production of numerous inflammatory cytokines, targeting of MerTK and possibly other TAM receptors therefore has the potential for inhibiting inflammatory cytokine production. Interestingly, the precipitation of a severe autoimmune phenotype in mice deficient in TAM receptor expression suggests that they may play a role in induction of suppressive macrophages (25). Therefore as briefly outlined above, unlike necrosis, not only does apoptosis not elicit an inflammatory response, it has immunomodulatory effects that are exerted through leukocytes such as APCs, regulatory cells and soluble factors as described further and illustrated in **Figure 1**.

### Soluble Factors

Apoptotic cells themselves release soluble mediators in their local milieu such as IL-10, TGF-β, and annexin A1 which exert immunosuppressive effects (26–28). In addition to that, macrophages that interact with apoptotic cells also downregulate immune response through release of IL-10, TGF-β and PGE_2_ together with a reduction in inflammatory cytokines such as IL-1α, IL-1β, IL-6, IL-12p70 and TNF-α (29–31). The downstream effects of these cytokines include but are not limited to the prevention of differentiation of T helper type 1 (Th1) and repression of MHC-II and costimulatory molecule expression on APCs. This deters further antigen presentation and T cell activation.

The release of TGFβ *in vitro* has been demonstrated to be carried out by recipient macrophages ingesting apoptotic cells but not during any other type of phagocytosis (32). This production is due mainly due to the ligation of PtdSers exposed on apoptotic cells to their receptor expressed on macrophages (32, 33). TGFβ induces T_regs_ (identified by expression of CD4^+^CD25^+^CD45RB^low^ CD62L^high^ intracellular CTLA-4^high^ and high forkhead-box transcription factor p3 (Foxp3) mRNA) in both peripheral blood and spleen in murine bone marrow transplantation model receiving apoptotic cell infusions (34). This effect on T_regs_ was not seen with TGF-β neutralization. This process is functionally relevant as well, wherein depletion of these T cells results in an augmented allogenic response.

IL-10 specifically is an anti-inflammatory cytokine that plays a role in tolerance induction and suppression of DC maturation (35). However, conclusive evidence linking apoptotic cell-induced suppression of adaptive immune responses exerted through IL-10 is lacking. This suggests that the mechanistic expanse of the immune responses to apoptotic cells likely extends beyond solely cytokine-mediated effects. Verbovetski et al. also outlined a role of complements in this process by demonstrating that uptake of iC3b-opsonized apoptotic cells resulted in upregulation of the expression of CCR7 on immature DCs, rendering these cells capable of migrating in response to CCR7 ligands to secondary lymphoid organs to initiate or maintain T cell peripheral tolerance (36).

### Control of APC Functions

Investigations of the effect of apoptotic cells on APCs have shown that ingestion of apoptotic cells by immature DCs leads to their resistance to maturation and activation, therefore inhibition of MHC Class II, CD40 and CD80/86 (37, 38). This in turn can decrease their ability to stimulate T cells despite intact apoptotic cell-derived antigen presentation. Effector T helper 17 (Th17) cells are suppressed while T_regs_ are induced through ingestion of apoptotic cells by DCs and the subsequent DC-T cell interaction in the presence of altered co-stimulatory and coinhibitory signals (39). Antigen coupled apoptotic cells induce T cell tolerance *via* IL-10 production and upregulation of PD-1 expression on APCs (40). PD-L1 on APCs then binds to CD80 expressed on T cells with a greater affinity than CD28 binding, and negatively regulates T cell activation (41).

One could hypothesize that macrophages contribute significantly to the tolerogenic response given that they induce T_regs_ (42). Supporting that hypothesis, various studies show the essential nature of macrophages in settings of tumor and autoimmune disease-related tolerogenic responses to apoptotic cells (43, 44).

Beyond macrophages and DCs, another distinct cell population that has been shown to play a role in apoptotic cell related immunosuppressive effect are monocytic-like (CD11b^+^Ly6C^high^) and granulocytic-like (CD11b^+^Gr1^high^) MDSCs (45). In cardiac allograft model, these cells exert their immunosuppressive effect by trafficking to the allograft where they inhibit local CD8 T cell accumulation and potentially induce and recruit T_regs_. Both populations have been shown to suppress T cell proliferation *in vitro* through antigen-dependent as well as antigen-independent methods *via* a variety of effector mechanisms, including nitric oxide (46), arginase, and reactive oxygen species (47–50). Furthermore, they promote T_reg_ induction through production of IL-10, TGF-β and indoleamine 2, 3-dioxygenase (51, 52). Most evidence suggests that MDSC subsets require IFN-γ, both for their induction and their effector function (53–56). Consequently, neutralization of IFN-γ completely abolishes the suppressive capacity of this population (57). For phagocytosis of apoptotic cells in the spleen, macrophages, T and natural killer (NK) cells are the potential sources of IFN-γ (58).

Another distinct APC population of interest is plasmacytoid dendritic cells (pDC). They have been not been demonstrated to be directly affected by apoptotic cells. However, the soluble factors released by macrophages upon interaction of with apoptotic cells can induce pDC activation, manifesting as an increased expression of CD86 and IFN-α (59). These pDCs can then stimulate T_reg_ generation through TGF-β dependent mechanisms. In cardiac allograft transplantation, alloantigen-presenting pDCs home to the lymph nodes in tolerogenic conditions, where they mediate alloantigen-specific T_reg_ cell development and prolong graft survival (60). Apoptotic cells can also drive activated pDCs to adopt a regulatory phenotype, capable of inducing IL-10-secreting T cells (61).

### Regulatory Cells

APCs are pivotal in priming T cell responses, but also in the induction of Foxp3^+^ T_regs_. This has been demonstrated after intravenous apoptotic cell infusions, local apoptotic death of epithelial cells and it occurs in a TGF-β dependent environment (62). Interestingly, the induced T_regs_ are likely antigen specific as was demonstrated in a murine arthritis model (63). The precise mechanisms that induce naïve T cell differentiation to T_regs_ requires further investigation however it’s distinctly clear that they play a vital role in maintenance of tolerance.

Apoptotic cells also activate splenic B cells to assume a regulatory phenotype which further induces CD4+ T cells to secrete IL-10. In a mouse collagen induced arthritis model, apoptotic cell therapy delayed the clinical onset and protected mice from severe joint inflammation and bone destruction through this mechanism where inhibition of IL-10 *in vivo* reversed the beneficial effects of apoptotic cells. These regulatory B cells (B_regs_) cells also produce IL-10 themselves and their passive transfer provides significant protection from arthritis to the mice (64).

## Apoptotic Cell Therapies in Solid Organ and Tissue Transplantation

As outlined above, apoptotic cells have the potential to be utilized in the field of transplantation due to their immunomodulatory potential and being a source of allo-antigens that can be captured and presented by APCs in an immunoquiescent environment. Intravenous infusion of apoptotic cells is the most commonly employed method of delivery. The use of donor derived apoptotic cells efficiently combines the delivery of apoptotic cells and donor antigens. However, provision of apoptotic signals and donor antigens can also be dissociated. For example, as outlined in the various studies described in the later part of this review, major histocompatibility complex (MHC) match between the apoptotic cells and the donor does not appear to be essential to induce tolerance in the recipient, as the delivery of any source of apoptotic cells (syngeneic, allogeneic, and xenogeneic) can induced recipient tolerance to the antigens co-delivered with the apoptotic cells. Therefore, while the source of apoptotic cells can be variable, the tolerance induced in this manner carries antigen-specificity that is established by the specific antigens provided at the time of apoptotic cell infusions (for example: apoptotic cells of donor origin; apoptotic cells infused with donor bone marrow cells; apoptotic cells infused to treat rejection or GVHD when donor cells are already present in the recipient). Once infused, these cells accumulate initially in the periphery of the splenic follicles within the marginal zone DCs and macrophages. Not only are apoptotic cells processed by recipient APCs to downregulate the indirect pathway T cells *via* negative co-stimulatory molecules, they can also directly interact with the direct pathway T cells and anergize these T cells by providing signal 1 without signal 2 (**Figure 1**) (65, 66).

Several *in vitro* methods can be utilized to induce apoptosis of cells. These include radiation strategies such as γ-radiation (65) or UV-B irradiation (66–68); and chemical treatments such as ethylene carbodiimide (ECDI) (69–72) or paraformaldehyde (73). An important consideration during the process of inducing apoptosis is to ensure early stage of cell apoptosis by the process, as late stages of apoptosis can in fact lead to immune activation due to loss of plasma membrane integrity, and subsequent release of intracellular contents and engagement of damage-associated molecular patterns (DAMPs) (74, 75). To determine the spectrum of stages from apoptosis to necrosis that the cells are in, one method is to quantify their surface annexin V and propidium iodine PI (PI) expression, wherein annexin V positivity marks apoptosis and PI positivity marks necrosis (72). The other important consideration is the timing of apoptotic cell infusion, most studies have achieved maximum benefit when infusions are administered 7 days prior to transplantation. This is likely to because it gives ample time for the processing of apoptotic cells by splenic APCs and subsequent induction of the aforementioned regulatory cell populations.

One of the effective methods that we have extensive experiences with and utilize to deliver donor apoptotic cells is through chemical treatment of donor splenocytes with ECDI (ECDI-SP) (71, 72, 76–78). ECDI is a hygroscopic, water-soluble chemical peptide cross-linker that acts by activating free carboxyl groups, catalyzing the formation of covalent peptide bonds between the active carboxyl group and primary amines (79, 80). The advantages of ECDI-treated cells are that they demonstrate better viability when maintained at 4°C, but within hours of *in vivo* administration they undergo rapid apoptosis (81). Cell based therapies such as donor specific transfusion (DST) carry a significantly higher risk of recipient sensitization, especially in those with pre-existing alloimmunity, while ECDI-SP might possibly confer therapeutic benefit in that scenario (82). In transplantation, ECDI treated cells have been used in non-human primates; while in autoimmune diseases, autoantigen-coupled syngeneic leukocytes have been developed for a phase I clinical trial for multiple sclerosis and have demonstrated the safety of this approach in this study (83).

Preclinical data from different groups has shown in murine models of cardiac transplantation that prolonged vascular allograft survival can be achieved through intravenous infusion of apoptotic donor splenocytes prior to transplantation. Sun et al. utilized UV or γ irradiation to induce apoptosis in splenocytes from donor strain rats, followed by confirming the apoptotic stage by using annexin V and PI staining (65). Apoptotic donor splenocytes were subsequently injected at a dose of 5 x 10^7^ per recipient a week prior to transplantation. This treatment alone resulted in a significant prolongation of graft survival from a median survival time of 7 days in untreated controls to 53 days in the treatment group. Histological analysis also revealed reduced leukocyte infiltration in the allograft in the treated recipients. Furthermore, the authors demonstrated that *in vivo* blockade of phagocytic activity prevented graft protection by this treatment. Another group led by Wang et al. independently tested the utility and mechanism of donor apoptotic cell infusions in a fully mismatched aortic allograft murine model (67). They established that donor apoptotic cell infusions downregulated indirect anti-donor response and improved chronic allograft vasculopathy (CAV). Through directly targeting DCs with allo-antigens, the anti-donor indirect T and B cell responses in allograft recipients were ameliorated. In liver transplantation in rats, donor apoptotic splenic lymphocytes have been shown to promote liver graft acceptance and increase peripheral T_regs_ as well (84, 85). Furthermore, in liver transplant rejection, administration of tolerogenic DCs with apoptotic lymphocytes alleviated the rejection while inducing immune tolerance (86).

Donor apoptotic cell infusions in islet transplantation in mice, have shown to prolong islet survival through T_reg_ induction and tolerogenic DCs (87, 88). Beyond murine studies, in non-human primates using donor apoptotic cell infusions have also shown promising results in allogeneic islet transplantation. An earlier study in non-human primates by Lei et al. showed prolonged islet allograft survival in monkeys infused with ECDI-SP on the day of transplantation; however, the effect was not sustained and the duration of graft survival following discontinuation of immunosuppression was 48 to 133 days, although the infusion of ECDI-SP was associated with significant CD4^+^CD25^+^Foxp3^+^ generation and expansion (89). Singh et al. used peri-transplant apoptotic donor leukocyte infusions, 7 days prior to transplant and 1 day after, along with short-term immunotherapy consisting of antagonistic anti-CD40 antibody, rapamycin, soluble tumor necrosis factor receptor, and anti-interleukin 6 receptor antibody for tolerance induction for intra-portal allogeneic islet transplantation in rhesus macaques (90). All of the five rhesus macaques showed operational tolerance to their islet allografts and demonstrated intact islets on histopathology of the liver at necropsy when the end point was reached. This strategy was successful in inducing long-term (≥1 year) tolerance of islet allografts in five of five non-sensitized, MHC class I-disparate, and one MHC class II DRB allele-matched rhesus macaques. Compared to monkeys that did not receive peri-transplant ECDI-SP infusions, the administration of ECDI-SP was associated with suppression of anti-donor CD4+ and CD8+ T effector memory (TEM) cell expansion within the circulating and liver mononuclear cells (LMNCs) and mesenteric lymph node (LNs). Additionally, a higher percentage of circulating natural suppressor and T_reg_ cells were present in the ECDI-SP-treated cohort. Notably, another cohort of fully MHC mismatched donor recipient pair did not show similar induction operational tolerance, or an increase in regulatory cell types or suppression of TEM responses. This could suggest that in this non-human primate study one-DRB-matched ECDI-SP infusion possibly provided a shared MHC II necessary for T_reg_ activation and/or expansion. Both studies demonstrate the overall safety of ECDI-treated leukocyte infusions, therefore providing a strong foundation for clinical translation of this approach (90).

To date, the only clinical trial utilizing a modified cell infusion for induction of transplant tolerance in solid organ transplant is a phase I trial of mitomycin-treated donor mononuclear cell infusions in ten kidney transplant recipients (91). The primary outcome of demonstrating safety of the infusions was achieved with the infusions being well tolerated without side effects. Importantly, none of the patients developed *de novo* donor specific antibodies (DSAs) or experienced any rejection episodes. The infusions were administered to three different subgroups of patients, in increment doses and at different time points with respect to their day of transplantation (group A: 1.5 x 10^6^ per kg body weight (BW) on day −2; group B: 1.5 x 10^8^ per kg BW on day −2 and group C: 1.5 x 10^8^ per kg BW on day −7). Interestingly, subsequent testing showed suppression of donor-stimulated recipient leukocyte proliferation, whereas response to third party stimulation was intact. The best results were observed with the higher dose given at the early (day −7) time point. The presence of a strong CD19^+^CD24^hi^CD38^hi^ B_reg_ induction together with IL-10 production and evidence of an immune tolerance signature similar to that seen in immune tolerance network studies (92) suggest that donor apoptotic cell infusions may promote donor-specific tolerance. This can be compared to the aforementioned similar IL-10 producing B_reg_ induction seen mice autoimmune disease model with apoptotic cell infusion treatment (64, 93).

Interestingly, the authors noted that infections caused a transient disappearance of donor-specific hypo-responsiveness as demonstrated by *in vitro* donor-stimulated recipient lymphocyte proliferation. This trial thus successfully demonstrated the safety and possible efficacy of donor apoptotic cells in inducing donor-specific hypo-responsiveness for solid organ transplantation.

## Apoptotic Cell Therapies in Bone Marrow Transplantation

MHC disparity between donor and recipient remains a challenge to HSCT. Presence of T cells of donor origin in the graft faciliates bone marrow engraftment and prevents disease relapse, however it can increase the risk of GVHD. Similarly, recipient T cells that are not eliminated during conditioning impairs bone marrow engraftment and increases the risk of disease relapse and graft failure. This constitutes a unique challenge with using T cell depletion strategies in bone marrow transplatantion(BMT) that result in T cell depletion of donor and recipient origins, therefore underscores the need for alternatives to global T cell depletion strategies in BMT.

In the last decade, due to their immunomodulatory effect, apoptotic cell therapies have entered clinical translation and been tested as a prophylactic therapy for acute GVHD in HLA-matched myeloablative allogenic BMT (94) (2). Notably, a phase I/IIa clinical trial enrolled 13 patients with hematological malignancies, and infused them with incremental doses of donor mononuclear apoptotic cells (ApoCell) on day −1 followed by BMT with a myeloablative conditioning regimen on day 0. Overall, six of the patients who received the higher dose of ApoCell showed 0% incidence of grade II to IV GVHD, and the remaining seven patients showed a lower incidence of GVHD compared to published data of historical controls not receiving ApoCell infusions. Notably, ApoCell infusions had no effect on the time to engraftment, chimerism, or incidence of infections among the treated subjects. These observations support the needs for larger trials with even higher doses and possibly more frequent dosing of ApoCells in BMT as a GVHD prophylaxis (94).

Bittencourt et al. evaluated the effect of administration of irradiated apoptotic leukocytes from either donor or non-donor sources in murine model of mismatched BMT to determine whether the source of the apoptotic cells had an effect on the outcome (68). The addition of apoptotic cells resulted in a significant increase in the number of engrafted mice, along with a higher percentage of donor type cells in the mice that received apoptotic splenocytes. Interestingly, this effect was indiscriminate of whether the injected apoptotic leukocytes were from third party or syngenic hosts, or even from xenogeneic hosts such as human blood mononuclear cells, suggesting that the MHC molecules of the apototic cells do not need to match to either the donor or the recipient for this approach to be effective. This study thus demonstrated that apoptotic cells could have a utility in overcoming MHC barriers in BMT through possibly cross-tolerizing anti-donor recipient T cells, and therefore may also be used to reduce the intensity of conditioning regimens (68). Donor and third party apoptotic cell infusions have shown to lower the incidence of donor allo‐immunization with only one out of forty-four mice developed DSA (95). This finding is in agreement with the reported poor immunogenicity of apoptotic cells compared with identical viable or non-replicating cells.

## Role of Apoptosis in Extracorporeal Photopheresis

Extracorporeal photopheresis (ECP) refers the process of UV-A radiation of autologous mononuclear cells obtained *via* leukapheresis, followed by photosensitization with by 8-methoxypsoralen (8-MOP) and infusion back to the patient. ECP was initially used to treat patients with cutaneous T-cell lymphoma (CTCL), but its indications for use have now extended to other conditions such as GVHD (96), scleroderma (97), and solid organ transplantation (98–100). In a standard ECP treatment, usually only 10% of total blood circulating mononuclear cells are obtained and exposed to 8-MOP, and the susceptibility to ECP-induced apoptosis varies from cell type to cell type (101). The exact mechanisms of the therapeutic effect of ECP still remains to be elucidated, but in CTCL it has been described that the ingestion of apoptotic cells by APCs results in production of anti-tumor cells targeting malignant lymphoid cells (102). This explains its beneficial effect in CTCL, however its utility in GVHD is likely to be due to a wider scope of less well-defined immunomodulatory effects.

Gorgun et al. demonstrated a shift in the cytokine profile toward a Th2 response in patients who underwent ECP for GVHD treatment (103). Specifically, they demonstrated an increase of IL-4, IL-10 and TGF-β and a concurrent decrease of IL-12, IL-1, interferon-γ, and TNF-α. Furthermore, leukocyte proliferation assays using DCs from patients undergoing ECP showed decreased proliferation of antigen-stimulated autologous and allogeneic T cells. Circulating T_regs_ with ECP therapy suppressed proliferation of allogenic effector T cells and their IFN-γ secretion (104). The above described T cell responses have prompted its use together with conventional pharmacotherapy for the treatment of GVHD as well as acute rejection of cardiac allografts in humans (101, 105–110).

## Limitations

### Prior Sensitization

Transplant recipients with memory cells as a result of previous sensitizing events can be challenging to transplant as they mount a rapid and aggressive immune response compared to their non-sensitized counterparts, thereby increasing the risk for immediate graft loss (111–113). The presence of donor specific antibodies (DSAs) can also lead to an accelerated rejection through complement activation, resulting in endothelial damage in solid organ transplantation (113). Burns et al., demonstrated in a sensitized murine cardiac transplant model that memory B cells override the tolerogenic effect of donor-specific transfusions (DST) combined with co-stimulation blockade by anti-CD154. Furthermore, they also facilitate the priming of alloreactive T cells and thus, in the presence of DSAs, result in accelerated graft loss (82, 114). A similar concern may also exist for apoptotic donor cell infusions in the presence of DSAs.

On the other hand, when DSAs are at low or negligible levels, we have demonstrated in a sensitized murine islet transplant model that infusions of donor ECDI‐SP together with transient anti-CD154 and rapamycin are effective in early inhibition of alloreactive T and B memory cells, therefore protect islet allograft function. Analysis of donor-specific T memory cells in these recipients treated with this combination therapy showed almost a complete absence in the islet allograft as well as in draining lymph nodes. Memory B cells also met a similar fate in that in recipients treated with this combination therapy, their numbers in draining lymph nodes were also significantly suppressed. These findings correlated with superior islet allograft survival in these previously sensitized recipients. Thus, the use of donor EDCI-SP also shows promises for transplantation of sensitized recipients (115).

### Infection and Tolerance

Opportunistic infections and latent viral activation, such as CMV, pose a considerable challenge in transplantation overall. In the context of tolerance, many of the aforementioned authors have described both in murine models, non-human primates and phase I clinical trials, that infections can negatively impact tolerance induction. Such infections have also been demonstrated to be deleterious to the stability of donor-specific tolerance, thereby effecting long-term host alloreactivity and graft survival (116).

Of the common pathogens, cytomegalovirus (CMV) is a highly prevalent virus that causes a symptomatic infection that has been noted as an independent risk factor for the development of acute rejection (117). Our lab has demonstrated in a murine islet transplant model that acute murine-CMV(MCMV) infection alters MDSC differentiation, promoting maturation of immature myeloid cells to become inflammatory monocytes which subsequently prime alloreactive CD8 T cells that prevent the induction of tolerance (78). In mice where MCMV infection was introduced days after donor ECDI-SP infusions, it not only led to the disruption of tolerance otherwise induced by donor ECDI-SP infusions, but also resulted in accelerated rejection of a subsequent same-donor islet transplant as a consequence of anti-donor memory T cell response (118).

Other pathogens that have been studied include the gram-positive intracellular bacteria Listeria monocytogenes (Lm). Wang et al. demonstrated that a sublethal dose of Lm in a tolerized cardiac transplant mouse model resulted in rejection of the cardiac allograft in nearly 40% of the recipients, while an additional 30% showed a slowing of the heartbeat and an enlargement of the allograft with histological evidence of increasing lymphocytic infiltration (119). Furthermore, through analysis of the gene signature of tolerized *versus* rejecting mice, they noted that only partial restoration of the tolerized gene signature had occurred at day 30 post Lm injection. Notably, with resolution of infection, intra-graft T_reg_ percentage returned to the pre-infection level. This suggests that partial, but not complete, return of tolerance occurred with resolution of the infection. In this model, Wang et al. further noted that the disruptive effect of Lm was prevented by IFNαR gene deficiency in their cardiac and skin transplantation recipients. Conversely, administration of IFN-β even without Lm infection, shortened skin allograft survival. Supporting this finding, Young et al. showed that Lm infection induced a transient increase in circulating IL-6 and IFN-β and with recovery from the infection, these cytokines returned to baseline (120). These findings suggest a role of type-1 interferon in tolerance disruption in setting of a Lm infection.

The data outlined above emphasizes the need for therapies that maintain tolerance or restore complete tolerance in the setting of inadvertent microbial infections. The potential targets whose roles need to be further elucidated in this process include type-1 interferon, IL-6, and other inflammatory cytokines.

The other facet relating donor-specific tolerance to risk of opportunistic infections is the potential of tolerance to minimize allograft inflammation and eliminate chronic immunosuppression, both of which may contribute to prevention of latent viral (e.g., CMV) reactivation, especially from the transplanted allograft. However, with the tolerance approach *via* bone marrow chimerism, aggressive conditioning regimens needed for BMT may in fact promote CMV reactivation, thus impairing bone marrow engraftment, and/or promoting subsequent loss of chimerism and tolerance (121, 122). These concerns again underscore that the alternative approach using apoptotic cell-based might be a more attractive option, taking into consideration of potential opportunistic infections particularly latent CMV reactivation.

## Summary and Future Directions

The profound immunoregulatory effects of donor apoptotic cells have been harnessed thus far in several murine and non-human primate experimental models where they have shown promising efficacy for transplant tolerance induction. Furthermore, recent early phase I/II clinical trials in both solid organ transplant and BMT have demonstrated the safety of this approach. As highlighted above, the major challenges with the use of apoptotic cell infusion include limitations in sensitized recipients and the loss of tolerance in setting of opportunistic microbial infections. Other potential obstacles include controlling for the early stage of apoptosis and the limited practicality of using donor apoptotic cells in diseased donor transplantation.

One pragmatic approach that can overcome logistical obstacles is the use of acellular carriers for solubilized donor antigens. This would obviate the need for procurement of a large number of donor cells, a particular logistical challenge in case of deceased donor transplantation. It can also make storage easier and ensure consistent quality in the manufacturing process. One such acellular carrier is polylactide-co-glycoside (PLG)-based nanoparticles. PLG nanoparticles can be coupled with membrane donor antigens, and in combination with a low dose rapamycin, have been shown to inhibit anti-donor response and prolong allograft survival as well as to prevent GVHD (123, 124). Furthermore, geometric modifications of PLG particles can modify cellular signaling networks and program them to alter subsequent immune cell activation therefore be utilized to create an immunoquiescent environment. Once such modification involves the presentation of phosphatidylserine which typically is expressed on the surface of apoptotic cells and may interact with phagocytic APC receptors. The subsequent signaling of this interaction likely through TGF-β production leads to activation of alloreactive T cells while promoting expansion of T_regs_ (125). Altogether, these data highlight the enormous potential of bioengineering the full immunomodulatory signaling program of apoptotic cells onto acellular carriers for the induction of transplant tolerance.

Promisingly, the future holds exciting potential for apoptotic cell therapy with its recent translation into clinical trials. However, a great deal remains to be learned of the underlying mechanisms together with methods to overcome its limitations when aiming for a more widespread clinical application.

## Author Contributions

IH and XL reviewed the literature relevant to the research topic. IH wrote the manuscript under consultation with XL. XL contributed to the critical revision of the article and the final approval of the version to be published. All authors contributed to the article and approved the submitted version.

## Conflict of Interest

The authors declare that the research was conducted in the absence of any commercial or financial relationships that could be construed as a potential conflict of interest.
